# Addition of immune checkpoint inhibitor to standard of care chemoradiotherapy in patients with locally advanced nasopharyngeal carcinoma: a systematic review and meta-analysis

**DOI:** 10.37349/etat.2026.1002403

**Published:** 2026-09-28

**Authors:** Ramaditya Srinivasmurthy, Jason Ta, Sharif Afifi, Taimur Khalid, Hazem Aboaid, Rishi Kumar Nanda, Daniel Thomas Jones, Kenny Do, Kevin Nguyen, Karina Fama, Sarah Aamer, Yin Mon Myat, Jo-Lawrence Bigcas, Kyaw Zin Thein

**Affiliations:** Fondazione Policlinico Universitario Agostino Gemelli IRCCS, Italy; ^1^Department of Internal Medicine, Mount Sinai Morningside, New York, NY 10019, USA; ^2^Department of Internal Medicine, HCA Healthcare, HCA Florida Citrus Hospital, Inverness, FL 34452, USA; ^3^Department of Internal Medicine, Kirk Kerkorian School of Medicine, University of Nevada, Las Vegas, NV 89154, USA; ^4^Department of Otolaryngology, Kirk Kerkorian School of Medicine, University of Nevada, Las Vegas, NV 89154, USA; ^5^College of Osteopathic Medicine, Touro University Nevada, Henderson, NV 89014, USA; ^6^Department of Internal Medicine, Sunrise Health GME Consortium, HCA Healthcare, Las Vegas, NV 89128, USA; ^7^Department of Internal Medicine, Interfaith Medical Center Campus, One Brooklyn Health, Brooklyn, NY 11213, USA; ^8^Division of Medical Oncology, Comprehensive Cancer Centers of Nevada, Las Vegas, NV 89169, USA

**Keywords:** immune-checkpoint-inhibitor, sintilimab, toripalimab, locally-advanced, nasopharyngeal-carcinoma, PFS, adverse events

## Abstract

**Background:**

Preliminary findings from this study were previously published as a conference abstract (https://doi.org/10.1158/2326-6074.IO2025-B039). Immune checkpoint inhibitors (ICIs) have shown activity in nasopharyngeal carcinoma (NPC), but their efficacy and safety when integrated with standard chemoradiotherapy (CRT) for locally advanced NPC remain under investigation. We performed a systematic review and meta-analysis of randomized controlled trials evaluating the efficacy and safety of integrating ICIs with standard CRT-based treatment in patients with locally advanced NPC.

**Methods:**

MEDLINE and EMBASE were searched from inception through September 1, 2025, for English-language phase II or III randomized controlled trials evaluating ICIs plus standard therapy. Eligible studies reported efficacy outcomes, including progression-free/event-free survival (PFS/EFS), overall survival (OS), distant metastasis-free survival (DMFS), and locoregional recurrence-free survival (LRFS), and/or safety outcomes. Hazard ratios (HRs) were pooled using the generic inverse variance method, and risk ratios (RRs) for adverse events were pooled using the Mantel-Haenszel method. Fixed-effect models were used, with heterogeneity assessed using *I*^2^ statistics.

**Results:**

Three trials randomized 1,022 patients: CONTINUUM (*n* = 423), DIPPER (*n* = 450), and a phase II toripalimab trial (*n* = 149). The pooled safety population included 998 patients. ICIs significantly improved PFS/EFS [HR 0.54; 95% confidence interval (CI): 0.40–0.72; *P* < 0.0001], DMFS (HR 0.52; 95% CI: 0.36–0.75; *P* = 0.0005), and LRFS (HR 0.47; 95% CI: 0.31–0.71; *P* = 0.0003). OS favored the ICI arm but was not statistically significant (HR 0.77; 95% CI: 0.49–1.20; *P* = 0.24). ICIs increased immune related adverse events, whereas most CRT-associated locoregional and hematologic toxicities were not significantly increased.

**Discussion:**

Adding ICIs to standard CRT based therapy significantly improved disease control outcomes, including PFS/EFS, DMFS, and LRFS, without broadly worsening the overall CRT associated toxicity profile. Longer follow up is needed to determine whether these early disease control benefits translate into a statistically significant OS advantage.

## Introduction

Nasopharyngeal carcinoma (NPC) is a relatively rare malignancy, with an annual global incidence of fewer than 1 in 100,000 people. However, its prevalence is significantly higher in regions such as East and Southeast Asia [1]. The etiology of NPC involves a combination of genetic susceptibility, Epstein-Barr virus (EBV) infection, and environmental exposures, which together disrupt the mucosal epithelium and promote malignant transformation [2]. Standard treatment for NPC includes high-dose radiation therapy, often combined with chemotherapy—typically cisplatin and 5-fluorouracil or gemcitabine—for advanced stages [3].

In recent years, immune checkpoint inhibitors (ICIs) have been developed to enhance antitumor immunity by blocking inhibitory pathways such as PD-1/PD-L1, thereby counteracting tumor immune evasion mechanisms [4]. ICIs have demonstrated efficacy across several malignancies—including melanoma, non-small cell lung cancer, and NPC—resulting in improved patient outcomes [5, 6].

Given the emergence of multiple randomized trials evaluating the integration of ICIs into chemoradiotherapy (CRT)-based treatment for locally advanced (LA) NPC [7, 8], a quantitative synthesis is needed to clarify the magnitude and consistency of benefit across clinically relevant endpoints. Individual trials may be underpowered to detect differences in survival outcomes such as OS, particularly in the setting of relatively favorable baseline outcomes and limited follow-up duration. In addition, uncommon but clinically meaningful adverse events, including high grade immune related adverse events (irAEs) and selected locoregional toxicities, may be difficult to interpret within individual trials because of low event rates. Therefore, we conducted a systematic review and meta-analysis of randomized controlled trials (RCTs) to evaluate whether adding ICIs to standard CRT-based therapy improves disease-control and survival outcomes in LA NPC while more precisely characterizing the associated toxicity profile.

## Materials and methods

This systematic review and meta-analysis was conducted in accordance with the Cochrane Handbook for Systematic Reviews of Interventions and reported according to the Preferred Reporting Items for Systematic Reviews and Meta-Analyses (PRISMA) guidelines [9].

A comprehensive literature search was performed in MEDLINE and EMBASE from database inception through September 1, 2025. Search terms included combinations of terms related to NPC, LA or nonmetastatic disease, immune checkpoint inhibition, PD-1/PD-L1 blockade, and individual agents including sintilimab, toripalimab, and camrelizumab. The full search strategies for each database are provided in the Supplementary materials. The search was limited to human, English-language RCTs in phase II or III settings. Non-randomized studies, trials in metastatic disease, and studies lacking relevant outcome data were excluded.

Study identification, screening, eligibility assessment, and inclusion were documented using a PRISMA flow diagram (Figure 1). After removal of duplicates, titles and abstracts were screened, followed by full-text review of potentially eligible studies. Studies were eligible if they evaluated the addition of an ICI to standard CRT-based treatment in patients with LA NPC and reported at least one relevant efficacy or safety outcome. Data extraction was performed independently by two reviewers (R.S and J.T), with discrepancies resolved by D.T.J. and H.A. Extracted data included trial design, patient characteristics, treatment protocols, and outcomes.

**Figure 1:**
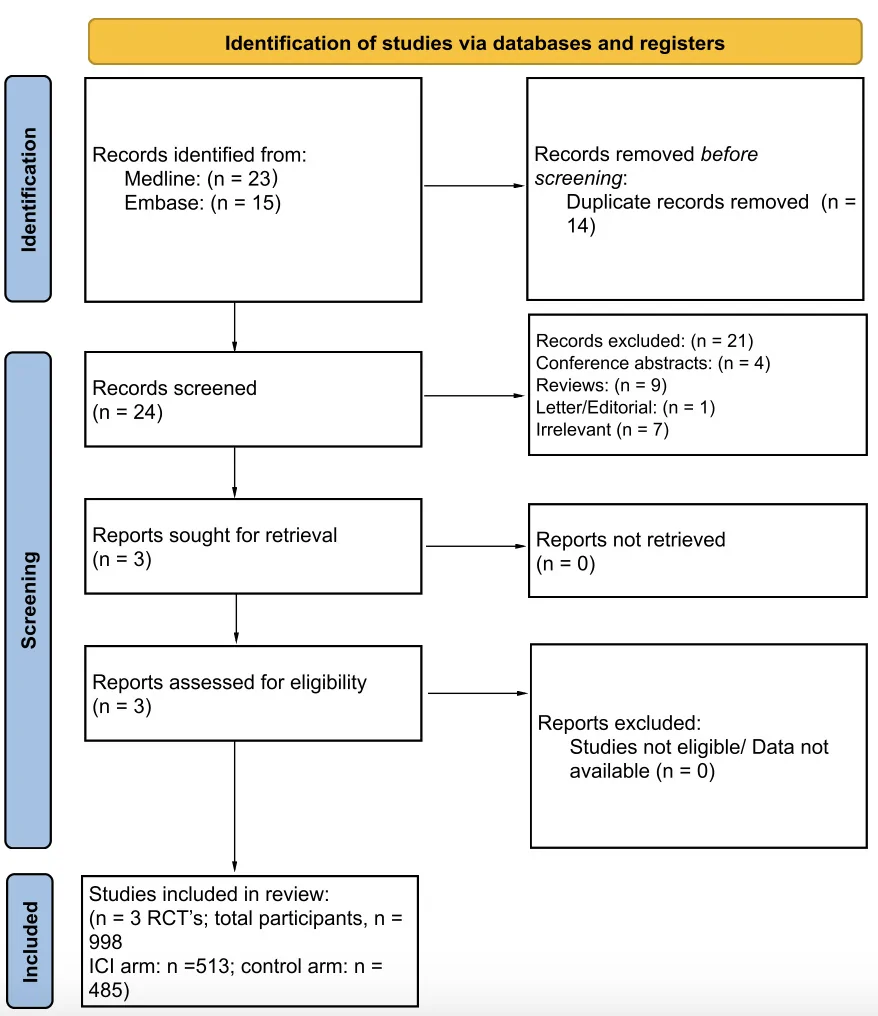
**Study flow diagram in accordance with Preferred Reporting Items for Systematic Reviews and Meta-Analyses (PRISMA) guidelines. Three randomized controlled trials were included in the quantitative synthesis, comprising 998 total participants, including 513 patients assigned to immune checkpoint inhibitor (ICI)-containing treatment arms and 485 assigned to control arms.** Adapted from [9]. © 2021, The Author(s). Distributed under a Creative Commons Attribution license (CC BY 4.0).

The primary objective was to evaluate pooled hazard ratios (HRs) with 95% confidence intervals (CIs) for progression-free/event-free survival (PFS/EFS), overall survival (OS), distant metastasis-free survival (DMFS), and locoregional recurrence-free survival (LRFS) in patients with LA NPC receiving ICIs in addition to standard treatment. Adverse events, including irAEs and hematologic toxicities, were also analyzed.

For time-to-event efficacy outcomes, including PFS/EFS, OS, DMFS, and LRFS, pooled HRs with 95% CIs were calculated using the generic inverse variance method. For dichotomous safety outcomes, including immune-related, locoregional, general, and hematologic adverse events, pooled risk ratios (RRs) with 95% CIs were calculated using the Mantel-Haenszel method. Fixed-effect models were used for the primary analyses because the included trials were RCTs evaluating the addition of PD-1 blockade to CRT-based standard therapy in the same disease setting of LA NPC. Heterogeneity was assessed using Cochran’s *Q* test and *I*^2^ statistics, with *I*^2^ > 50% considered to indicate substantial heterogeneity. Outcome-specific pooled analyses were performed for individual efficacy and safety endpoints; these analyses were not considered formal subgroup analyses. Statistical significance was defined as *P* < 0.05. Funnel plots were generated for exploratory assessment of publication bias, although interpretation was limited by the small number of included studies; risk of bias was assessed using the Cochrane risk-of-bias tool (Figures 2 and 3, respectively). Analyses were performed using Cochrane RevMan software (v5.3).

**Figure 2:**
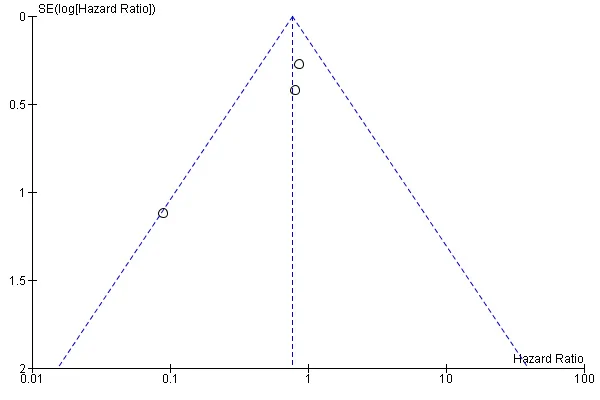
**Funnel plot evaluating potential publication bias for survival outcomes.** Funnel plot of included studies assessing the effect of immunotherapy in locoregionally advanced nasopharyngeal carcinoma. Each circle represents one study. The x-axis represents the hazard ratio on a logarithmic scale, and the y-axis represents the standard error of the log hazard ratio. Visual inspection was limited by the small number of included studies.

**Figure 3:**
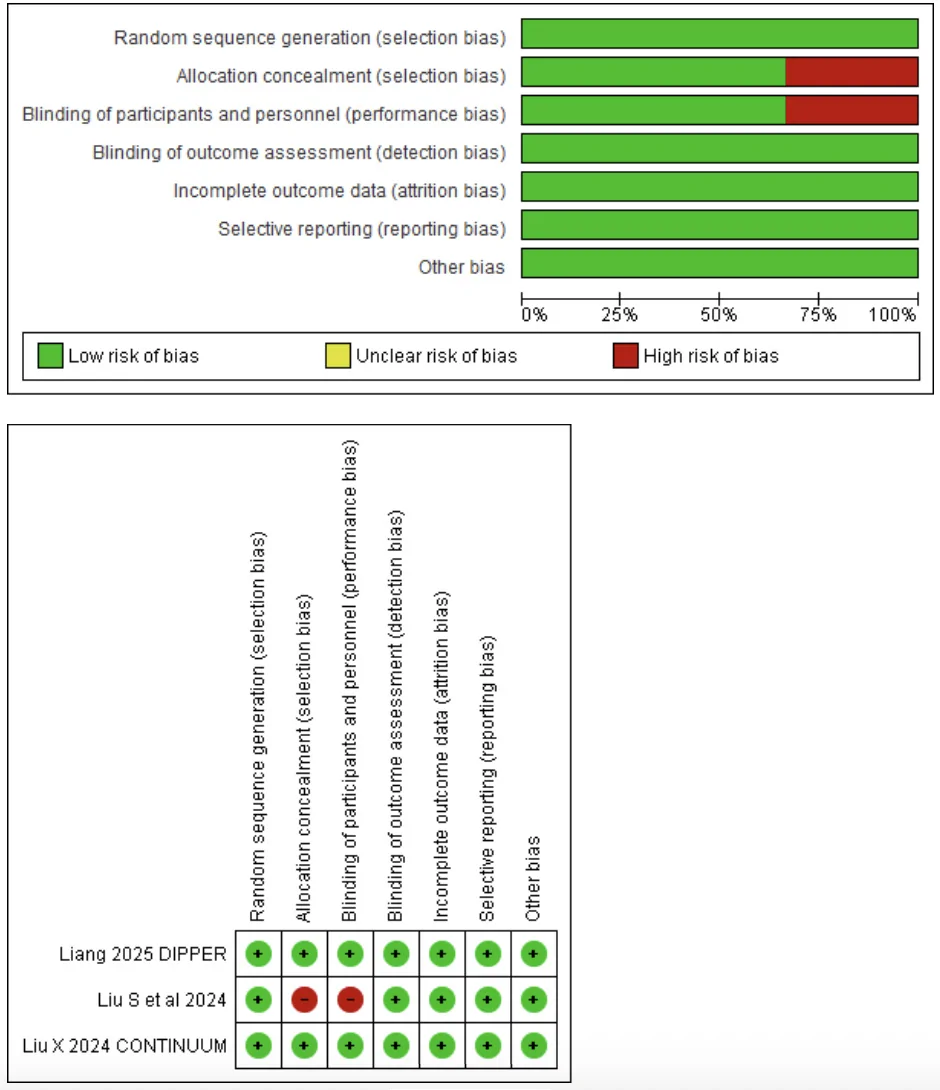
**Risk of bias graph for included randomized controlled trials.** Summary of review authors’ judgments for each risk-of-bias domain across all included studies, presented as percentages. Domains assessed included random sequence generation, allocation concealment, blinding of participants and personnel, blinding of outcome assessment, incomplete outcome data, selective reporting, and other sources of bias.

## Results

Three RCTs comprising 1,022 total patients met the inclusion criteria and were included in the quantitative synthesis: the phase III CONTINUUM trial (*n* = 423) [10], the phase III DIPPER trial (*n* = 450) [11], and a randomized phase II toripalimab trial (*n* = 149) [12]. Across all studies, 513 patients were assigned to ICI-containing treatment arms, and 485 patients were assigned to control arms. Trial characteristics are summarized in Table 1.

**Table 1:** Characteristics of the studies included in the meta-analysis.

**Study (author/year)**	**Study type**	**Number of patients [immune checkpoint inhibitor (ICI) group/control]**	**Cancer type**	**Treatment regimen**	**Primary outcome measure**
**CONTINUUM**	Phase III, randomized controlled trial (RCT)	423 total; 209 ICI/214 control	Locally advanced nasopharyngeal carcinoma (LA NPC)	Chemotherapy induction (gemcitabine + cisplatin) followed by cisplatin radiotherapy versus standard + sintilimab (ICI)	Event-free survival
**DIPPER**	Phase III, RCT	450 randomized (226 ICI/224 control); 426 included in safety analysis (205 ICI/221 control)	LA NPC	Chemotherapy induction + concurrent chemotherapy, then camrelizumab versus observation	Event-free survival
**Phase II toripalimab trial**	Phase II, RCT	149 total; 99 ICI/50 control	LA NPC	Neoadjuvant toripalimab versus placebo, followed by concurrent chemoradiotherapy, then adjuvant toripalimab versus placebo	Progression-free survival
**Total**	-	998 total; 513 ICI/485 control	-	-	-

The CONTINUUM trial compared chemotherapy induction with gemcitabine and cisplatin followed by cisplatin CRT (control group) versus standard therapy with the addition of sintilimab (treatment group) in a 1:1 randomization ratio. The DIPPER trial randomized patients (1:1) who had completed induction and concurrent CRT to receive either adjuvant camrelizumab (treatment group) or observation (control group). The phase II trial compared the addition of neoadjuvant toripalimab (treatment) versus placebo (control) in a 2:1 randomization ratio, followed by concurrent CRT followed by adjuvant toripalimab versus placebo. Across all included studies, 513 patients were assigned to ICI-containing treatment arms, and 485 patients were assigned to control arms. Heterogeneity for irAE outcomes ranged from *I*^2^ = 0% to 97%, with outcome-specific *I*^2^ values reported alongside each pooled estimate in the corresponding forest plots.

Risk of bias and publication bias assessments are summarized in Figures 2 and 3. Overall, the included studies were RCTs with generally acceptable methodological quality based on the Cochrane risk-of-bias tool. Several domains were limited by differences in study design, including open-label treatment allocation in some trials and variation in blinding procedures. Visual inspection of the funnel plot did not demonstrate clear asymmetry; however, interpretation was limited by the small number of included studies.

Our analysis showed a significantly improved PFS/EFS (2 years in the Phase II trial and 3 years in CONTINUUM and DIPPER trial) in the ICI arm compared to the control arm, with a 46% reduction in risk of progression and an HR of 0.54 (95% CI: 0.40–0.72, *P* < 0.0001; *I*^2^ = 0%) (Figure 4A). The DMFS was improved in the ICI treatment arm compared to the control arm, yielding a risk reduction of events of 48% with an HR of 0.52 (95% CI: 0.36–0.75, *P* = 0.0005; *I*^2^ = 0%) (Figure 4B). The LRFS was also significantly improved in the ICI arm compared to the control arm, with a risk reduction of events of 53% and an HR of 0.47 (95% CI: 0.31–0.71, *P* = 0.0003; *I*^2^ = 60%) (Figure 4C). In contrast, the difference in OS between the treatment and control arm was not statistically significant; our data yielded a risk reduction of 23% with an HR of 0.77 (95% CI: 0.49–1.20, *P* = 0.24; *I*^2^ = 48%) (Figure 4D). However, further maturation of the data is required to draw definitive conclusions.

**Figure 4:**
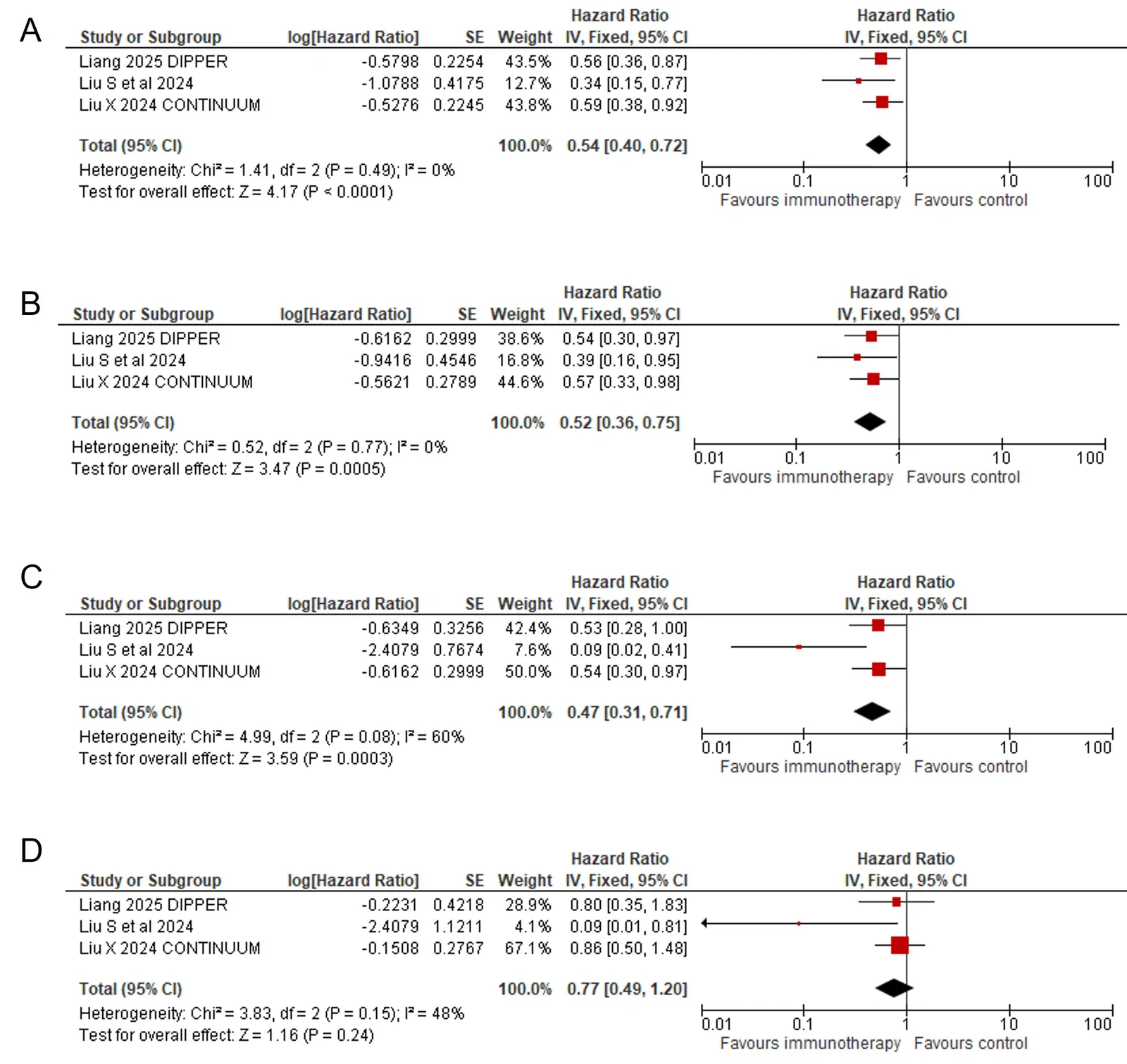
**Forest plots for efficacy outcomes.** (**A**) Progression-free survival or event-free survival; (**B**) distant metastasis-free survival; (**C**) locoregional recurrence-free survival; and (**D**) overall survival. CI: confidence interval.

We also analyzed the incidence of irAEs (Figure 5). The incidence of any grade irAEs was reported in 71.2% of patients in the ICI treatment arm versus 2.3% in the control arm, yielding an RR of 22.92 (95% CI: 13.99–37.57, *P* < 0.00001; *I*^2^ = 97%) (Figure 5A). High grade irAEs also occurred more frequently in the treatment arm with a reported incidence of 7.8% versus 0% in the control arm with an RR of 23.62 (95% CI: 4.83–115.49, *P* < 0.0001; *I*^2^ = 0%) (Figure 5B). The incidence of grade 1 or 2 hypothyroidism was 29.0% in the ICI treatment arm versus 9.5% in the control arm with an RR of 3.04 (95% CI: 2.24–4.11, *P* < 0.00001; *I*^2^ = 88%) (Figure 5C). In terms of dermatologic issues, the incidence of both any grade and high grade rash was higher in the ICI arm versus the control arm. Any grade rash was reported in 17.3% of patients in the treatment arm versus 0.8% in the control arm with an RR of 15.57 (95% CI: 6.55–36.98, *P* < 0.00001; *I*^2^ = 79%) (Figure 5D). The incidence of high grade rash was 3.3% in the treatment arm versus 0% in the control arm with an RR of 13.28 (95% CI: 1.86–94.98, *P* = 0.010; *I*^2^ = 0%) (Figure 5E). The incidence of any grade pruritus was also statistically significantly higher in the treatment arm versus control arm, occurring in 20.3% and 1.4%, respectively (RR of 12.29; 95% CI: 5.96–25.34, *P* < 0.00001; *I*^2^ = 65%) (Figure 5F). In contrast, the incidence of high grade pruritus was not statistically significant, with an incidence of 1.2% in the treatment arm and 0% in the control arm (RR of 5.69; 95% CI: 0.79–41.09, *P* = 0.08; *I*^2^ = 0%) (Figure 5G). The incidence of any grade allergic reaction was higher in the treatment arm (2.3%) versus the control arm (0%) with an RR of 7.66 (95% CI: 1.48–39.71, *P* = 0.02; *I*^2^ = 0%) (Figure 5H). The incidence of high grade allergic reaction was not statistically significant with an incidence of 0.4% in the treatment arm versus 0% in the control arm (RR: 2.19; 95% CI: 0.24–20.20, *P* = 0.49; *I*^2^ = 0%) (Figure 5I).

**Figure 5:**
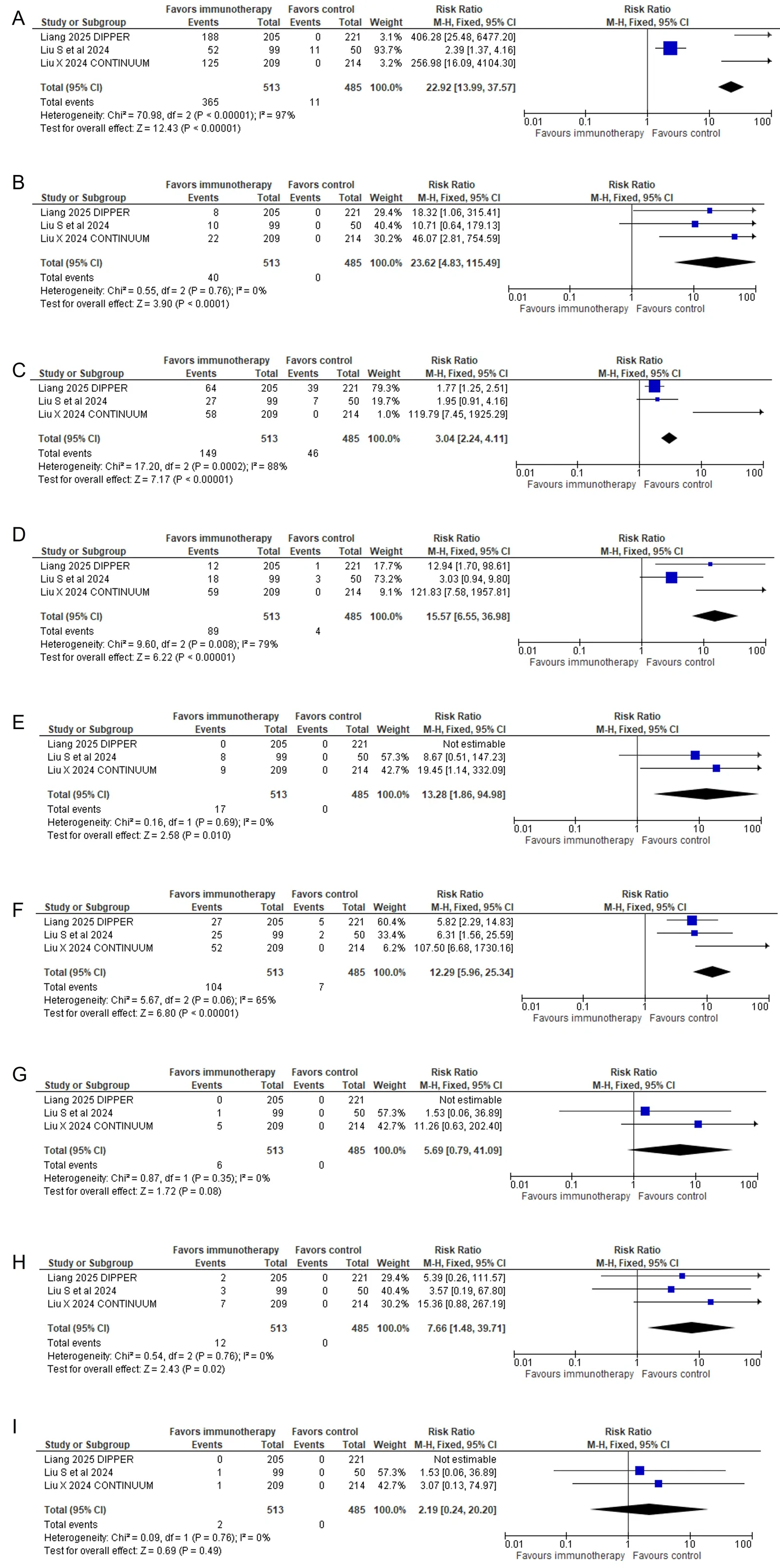
**Forest plots for immune related adverse events (irAEs).** (**A**, **B**) Any grade and high grade irAEs; (**C**) grade 1–2 hypothyroidism; (**D**, **E**) any grade and high grade rash; (**F**, **G**) any grade and high grade pruritus; and (**H**, **I**) any grade and high grade allergic reaction. CI: confidence interval.

Our data also pooled the incidence of locoregional adverse events (Figure 6) and general/hematological adverse events (Figure 7) between the treatment and control arms.

**Figure 6:**
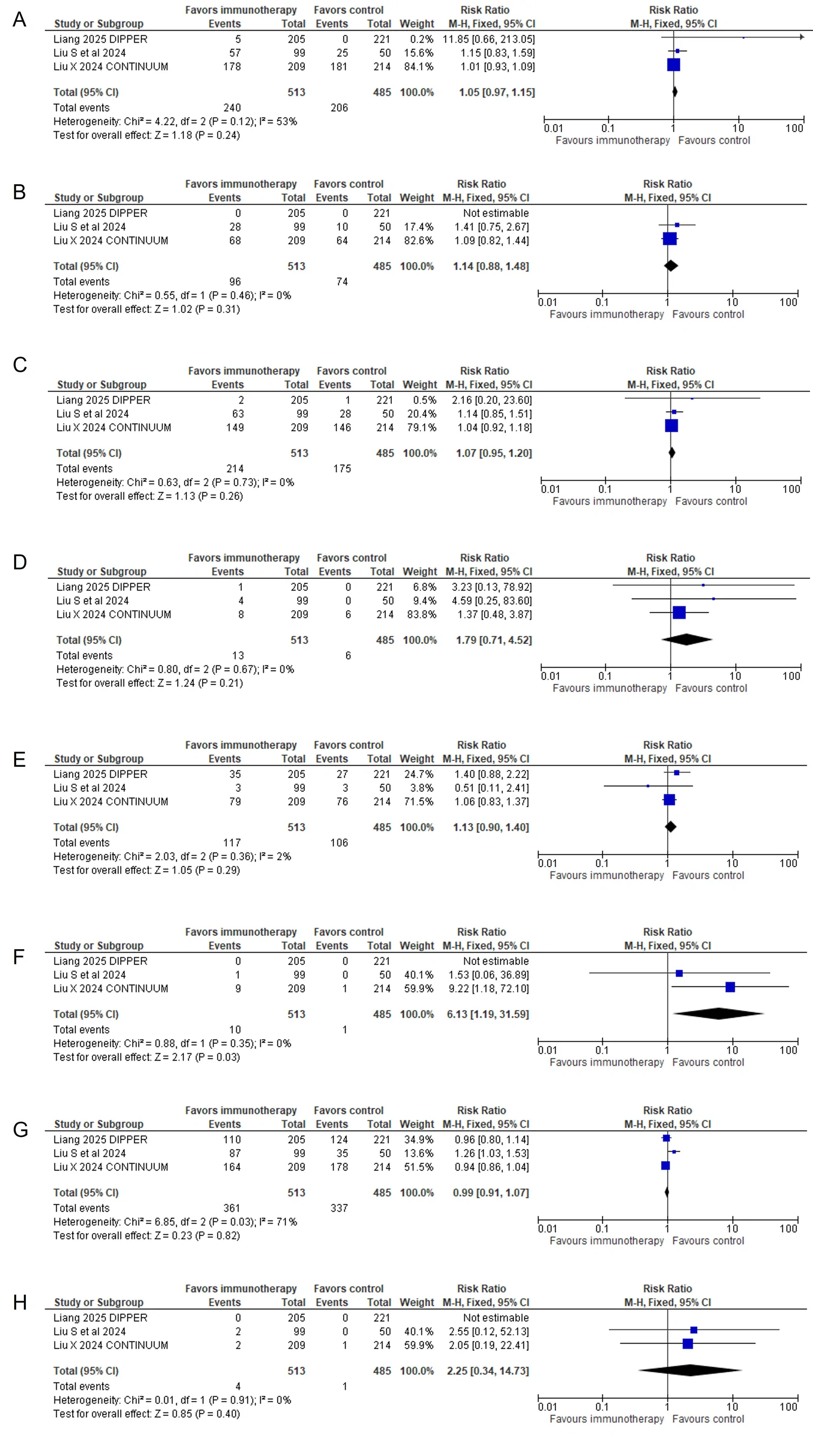
**Forest plots for locoregional adverse events.** (**A**, **B**) Any grade and high grade mucositis; (**C**, **D**) any grade and high grade dermatitis; (**E**, **F**) any grade and high grade dysphagia; and (**G**, **H**) any grade and high grade dry mouth. CI: confidence interval.

**Figure 7:**
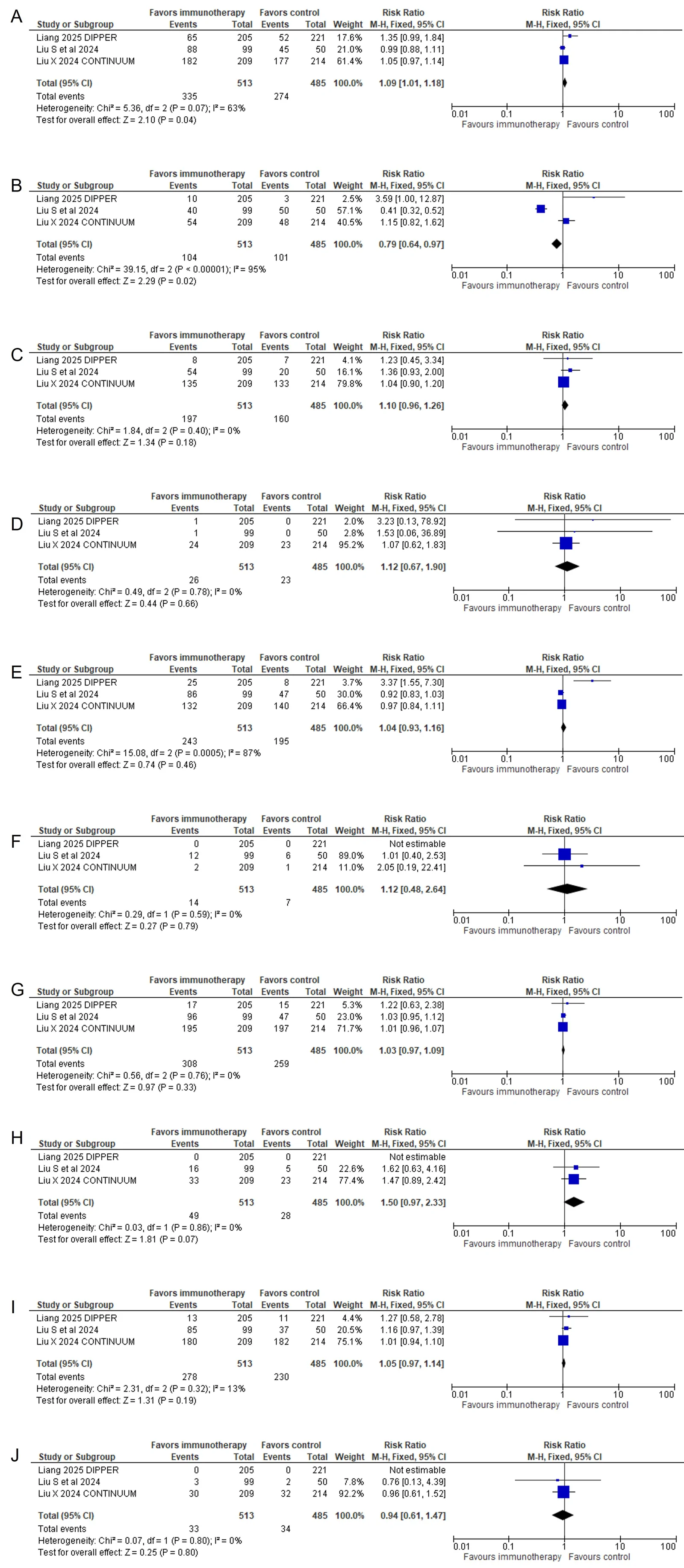
**Forest plots for general and hematological adverse events.** (**A**, **B**) Any grade and high grade leukopenia; (**C**, **D**) any grade and high grade vomiting; (**E**, **F**) any grade and high grade weight loss; (**G**, **H**) any grade and high grade anemia; and (**I**, **J**) any grade and high grade nausea. CI: confidence interval.

In terms of locoregional effects, the incidence of any grade and high grade mucositis was 46.8% and 18.7% in the treatment arm versus 42.5% and 15.3% in the control arm yielding an RR of 1.05 (95% CI: 0.97–1.15, *P* = 0.24; *I*^2^ = 53%) (Figure 6A) and 1.14 (95% CI: 0.88–1.48, *P* = 0.31; *I*^2^ = 0%) (Figure 6B) respectively however this failed to reach statistical significance. The incidence of any and high grade dermatitis also failed to reach statistical significance with an incidence of 41.7% and 2.5% in the treatment arm versus 36.1% and 1.2% in the control arm with an RR of 1.07 (95% CI: 0.95–1.20, *P* = 0.26; *I*^2^ = 0%) (Figure 6C) and 1.79 (95% CI: 0.71–4.52, *P* = 0.21; *I*^2^ = 0%) (Figure 6D) respectively. In contrast, although incidence of any grade dysphagia failed to reach statistical significance [22.8% in the treatment arm versus 21.9% in the control arm, RR of 1.13, 95% CI: 0.90–1.40, *P* = 0.29; *I*^2^ = 2% (Figure 6E)], the incidence of high grade dysphagia between the treatment and control arms were statistically significant, with an incidence of 2.0% in the treatment arm versus 0.2% in the control arm, RR of 6.13 (95% CI: 1.19–31.59, *P* = 0.03; *I*^2^ = 0%) (Figure 6F). Lastly the incidence of any and high grade dry mouth in the treatment arm was 70.4% and 0.8% in the treatment arm versus 69.5% and 0.2% in the control arm with an RR of 0.99 (95% CI: 0.91–1.07, *P* = 0.82; *I*^2^ = 71%) (Figure 6G) and 2.25 (95% CI: 0.34–14.73, *P* = 0.40; *I*^2^ = 0%) (Figure 6H) respectively.

Regarding general and hematological adverse events, the only statistically significant findings were those of any grade and high grade leukopenia. The incidence of any grade leukopenia was 65.3% in the treatment arm compared to 56.5% in the control arm with an RR of 1.09 (95% CI: 1.01–1.18, *P* = 0.04; *I*^2^ = 63%) (Figure 7A). In contrast, the incidence of high grade leukopenia was actually lower in the treatment arm (20.3%) in the treatment arm versus the control arm (20.8%) with an RR of 0.79 (95% CI: 0.64–0.97, *P* = 0.02; *I*^2^ = 95%) (Figure 7B).

The incidences of any and high grade vomiting were 38.4% and 5.1% in the treatment arm versus 33.0% and 4.7% in the control arm, with RR of 1.10 [95% CI: 0.96–1.26, *P* = 0.18 *I*^2^ = 0% (Figure 7C)] and 1.12 [95% CI: 0.67–1.90, *P* = 0.66; *I*^2^ = 0% (Figure 7D)] respectively. The incidences of any and high grade weight loss were 47.4% and 2.7% in the treatment arm versus 40.2% and 1.4% in the control arm with RR of 1.04 (95% CI: 0.93–1.16, *P* = 0.46; *I*^2^ = 87%) (Figure 7E) and 1.12 (95% CI: 0.48–2.64, *P* = 0.79; *I*^2^ = 0%) (Figure 7F). The incidences of any and high grade anemia were 60.0% and 9.6% in the treatment arm versus 53.4% and 5.8% in the control arm with RR of 1.03 (95% CI: 0.97–1.09, *P* = 0.33; *I*^2^ = 0%) (Figure 7G) and 1.50 (95% CI: 0.97–2.33, *P* = 0.07; *I*^2^ = 0%) (Figure 7H) respectively. The incidences of any grade and high grade nausea were 54.2% and 6.4% in the treatment arm versus 47.4% and 7.0% in the control arm with RR of 1.05 (95% CI: 0.97–1.14, *P* = 0.19; *I*^2^ = 13%) (Figure 7I) and 0.94 (95% CI: 0.61–1.47, *P* = 0.80; *I*^2^ = 0%) (Figure 7J) respectively.

## Discussion

Despite a five-year survival rate of approximately 80% with standard CRT, patients with LA NPC face a substantial risk of recurrence and metastasis, highlighting the need for improved therapeutic strategies [13]. ICIs have emerged as promising agents in this space, targeting immune evasion mechanisms and enhancing anti-tumor responses [13].

Our meta-analysis demonstrated that adding ICIs to CRT significantly improved PFS/EFS, DMFS, and LRFS, with risk reductions of 46%, 48%, and 53%, respectively. Although the pooled OS estimate favored the ICI-containing arms, with a 23% relative reduction in the risk of death, this difference did not reach statistical significance (HR 0.77; 95% CI: 0.49–1.20; *P* = 0.24). This likely reflects, at least in part, the limited maturity of the available survival data. Both phase III trials primarily reported 3-year outcomes, with median follow-up of approximately 42 months in CONTINUUM and 39 months in DIPPER, while the phase II toripalimab trial used 2-year PFS as its primary endpoint. In LA NPC, where modern CRT-based regimens already achieve relatively favorable survival outcomes, OS differences may require longer observation and a larger number of death events to become detectable. In contrast, endpoints such as PFS/EFS, DMFS, and LRFS are expected to mature earlier and may better capture early disease-control benefits from adding PD-1 blockade. Therefore, the absence of a statistically significant OS benefit in the current analysis should not be interpreted as a lack of survival effect, but rather as an immature endpoint requiring longer follow-up.

These findings are consistent with prior studies demonstrating improved objective response rates and tolerability of ICIs in LA NPC, particularly agents targeting the PD-1/PD-L1 axis [13, 14]. Although all ICIs included in this analysis target the PD-1/PD-L1 axis, pharmacologic differences among sintilimab, toripalimab, and camrelizumab may partly contribute to variability in efficacy and toxicity outcomes across trials. Sintilimab is a fully human IgG4 anti-PD-1 antibody that blocks PD-1 interaction with PD-L1 and PD-L2, with epitope mapping studies identifying key binding residues in the PD-1 FG loop that overlap with the PD-1/PD-L1 interaction [15, 16]. Toripalimab is a humanized anti-PD-1 antibody with a distinct FG-loop binding profile and high PD-1 binding affinity, which has been associated with enhanced T-cell activation and increased Th1/myeloid inflammatory cytokine responses in preclinical analyses [17]. Camrelizumab is also a humanized IgG4 anti-PD-1 antibody [18]. Structural studies indicate that N58 glycosylation of PD-1 promotes camrelizumab binding and that the antibody primarily engages PD-1 through its heavy chain [19]. Additionally, camrelizumab has been associated with distinctive dermatologic irAEs such as reactive cutaneous capillary endothelial proliferation [18, 20]. Therefore, while the pooled efficacy and safety findings support the overall benefit of PD-1 blockade in LA NPC, differences in antibody structure, epitope binding, Fc characteristics, and immune activation profiles may contribute to heterogeneity and could influence specific irAEs, including endocrine toxicities such as thyroid dysfunction [21]. Importantly, this meta-analysis was not designed or statistically powered to compare the efficacy or safety profiles of individual PD-1 inhibitors. Therefore, given the absence of head-to-head comparisons among these agents in LA NPC, these mechanistic considerations remain hypothesis-generating and should be explored in future comparative studies.

Our study also found an isolated increase in high grade dysphagia despite no significant increase in most other locoregional toxicities may reflect the vulnerability of the irradiated upper aerodigestive tract to overlapping inflammatory injury. Dysphagia after head and neck radiotherapy or CRT can result from acute mucosal inflammation, edema, pain, salivary dysfunction, and injury to pharyngeal swallowing structures [22, 23]. PD-1 blockade may further amplify local immune activation in previously or concurrently irradiated mucosa, potentially converting otherwise low-grade swallowing discomfort into clinically significant dysphagia in a small subset of patients [24]. However, this finding should be interpreted cautiously given the low absolute event rate, wide CI, and lack of a parallel statistically significant increase in any grade dysphagia, mucositis, dermatitis, or dry mouth. Therefore, the observed signal is hypothesis-generating and warrants confirmation in larger studies with standardized reporting of swallowing-related toxicities.

In terms of safety, irAEs were significantly more common in the ICI arms. Any grade irAEs occurred in 71.2% of patients receiving ICIs versus 2.3% in controls (RR 22.92, *P* < 0.00001), while high grade irAEs occurred in 7.8% versus 0%, respectively (RR 23.62; 95% CI: 4.83–115.49; *P* < 0.0001). Endocrine and dermatologic toxicities were notably increased, including grade 1–2 hypothyroidism (29.0% versus 9.5%, RR 3.04), any grade rash (17.3% versus 0.8%, RR 15.57), and high grade rash (3.3% versus 0%, RR 13.28). Most general, hematologic, and locoregional adverse events were not significantly increased, although isolated signals were observed for high grade dysphagia and leukopenia. This suggests that while ICIs heighten the risk of irAEs, they do not broadly increase toxicity when integrated with CRT. These findings are consistent with previous reports highlighting the risk of autoimmune-like complications associated with PD-1 inhibitors like sintilimab [25].

The mechanism of irAEs involves disruption of peripheral immune tolerance. PD-1 blockade leads to unchecked T-cell activation, increased pro-inflammatory cytokine production, and reduced regulatory T-cell function, resulting in immune-mediated damage to normal tissues [15, 25–27]. Common irAEs include rash, thyroiditis, adrenalitis, and colitis, with severity ranging from mild symptoms to life-threatening complications such as autoimmune myocarditis [26, 27]. Individual susceptibility to irAEs is still poorly understood but may relate to baseline immune status and genetic factors [28].

Management of irAEs requires a stratified approach. Grade 1 toxicities are typically managed with observation and supportive care, while grade 2 events often necessitate temporary treatment interruption and corticosteroids. Grade 3–4 toxicities require high-dose corticosteroids and sometimes additional immunosuppressive agents such as infliximab, mycophenolate mofetil, or intravenous immunoglobulin (IVIG) [29, 30]. Organ-specific interventions, such as hormone replacement or respiratory support, are critical for reducing morbidity.

This study has several limitations. First, the included trials utilized different ICIs—toripalimab in the phase II trial, camrelizumab in DIPPER, and sintilimab in CONTINUUM—and employed differing treatment sequences (neoadjuvant versus post-induction immunotherapy), which may affect efficacy and safety outcomes. Additionally, the trials used different randomization ratios (2:1 versus 1:1), potentially impacting balance between arms and statistical power. These methodological differences underscore the need for large, uniform phase III trials to confirm the role of ICIs in LA NPC.

Additionally, although MEDLINE and EMBASE were searched using expanded Boolean strategies, we did not include additional databases or trial registries such as the Cochrane Library, Web of Science, Scopus, or ClinicalTrials.gov, which may increase the risk of missing unpublished, ongoing, or recently completed studies. Future updates of this meta-analysis should incorporate these additional bibliographic databases and trial registries to improve search comprehensiveness and better capture emerging or unpublished randomized data.

### Conclusions

In this systematic review and meta-analysis of RCTs in LA NPC, the addition of ICIs to standard CRT-based treatment significantly improved PFS/EFS, DMFS, and LRFS, while OS remained immature and not statistically significant. In addition, our analysis highlights the safety profiles of ICI and standard of care combination regimens in LA NPC. The use of ICIs significantly increased the risk of any and high grade irAEs, any and high grade rash, grade 1 or 2 hypothyroidism, any grade pruritus, and any grade allergic reactions when compared to the control arm. Though these findings suggest additional toxicities with ICI use, close monitoring may facilitate early detection and allow for the initiation of appropriate supportive care, maintaining good patient outcomes and quality of life. Additionally, the use of ICIs along with standard therapy resulted in a similar safety profile with regard to general, hematological, and locoregional events compared to standard therapy alone. This suggests that with cautious and diligent administration, the therapeutic benefits of ICI use likely offset the risk of associated adverse events in patients with LA NPC.

## Abbreviations

CIs: confidence intervals
CRT: chemoradiotherapy
DMFS: distant metastasis-free survival
EBV: Epstein-Barr virus
HRs: hazard ratios
ICIs: immune checkpoint inhibitors
irAEs: immune related adverse events
LA: locally advanced
LRFS: locoregional recurrence-free survival
NPC: nasopharyngeal carcinoma
OS: overall survival
PFS/EFS: progression-free/event-free survival
PRISMA: Preferred Reporting Items for Systematic Reviews and Meta-Analyses
RCTs: randomized controlled trials
RRs: risk ratios

## Declarations

### Availability of data and materials

All relevant data are contained within the manuscript. All datasets analyzed for this study are included in the manuscript and the supplementary files. The datasets supporting the findings of this study are available from the corresponding author upon reasonable request.

### Copyright

© The Author(s) 2026.

## References

[1] Chang ET, Ye W, Zeng YX, Adami HO (2021). The Evolving Epidemiology of Nasopharyngeal Carcinoma. Cancer Epidemiol Biomark Prev.

[2] Tao Q, Chan AT (2007). Nasopharyngeal carcinoma: molecular pathogenesis and therapeutic developments. Expert Rev Mol Med.

[3] Nasopharyngeal Carcinoma Treatment (PDQ®)–Health Professional Version [Internet]. https://www.cancer.gov/types/head-and-neck/hp/adult/nasopharyngeal-treatment-pdq.

[4] Meng L, Wu H, Wu J, Ding P, He J, Sang M (2024). Mechanisms of immune checkpoint inhibitors: insights into the regulation of circular RNAS involved in cancer hallmarks. Cell Death Dis.

[5] Sun Q, Hong Z, Zhang C, Wang L, Han Z, Ma D (2023). Immune checkpoint therapy for solid tumours: clinical dilemmas and future trends. Signal Transduct Target Ther.

[6] American Cancer Society. Immunotherapy for nasopharyngeal cancer [Internet]. https://www.cancer.org/cancer/types/nasopharyngeal-cancer/treating/immunotherapy.html.

[7] American Cancer Society. Immune checkpoint inhibitors and their side effects [Internet]. https://www.cancer.org/cancer/managing-cancer/treatment-types/immunotherapy/immune-checkpoint-inhibitors.html.

[8] Mayo Clinic. Immune checkpoint inhibitors and thyroid dysfunction: a case from the endocrine teaching clinics [Internet]. https://www.mayoclinic.org/medical-professionals/endocrinology/news/immune-checkpoint-inhibitors-and-thyroid-dysfunction-a-case-from-the-endocrine-teaching-clinics/mcc-20439104.

[9] Page MJ, McKenzie JE, Bossuyt PM, Boutron I, Hoffmann TC, Mulrow CD (2021). The PRISMA 2020 statement: an updated guideline for reporting systematic reviews. BMJ.

[10] Liu X, Zhang Y, Yang KY, Zhang N, Jin F, Zou GR (2024). Induction-concurrent chemoradiotherapy with or without sintilimab in patients with locoregionally advanced nasopharyngeal carcinoma in China (CONTINUUM): a multicentre, open-label, parallel-group, randomised, controlled, phase 3 trial. Lancet.

[11] Liang YL, Liu X, Shen LF, Hu GY, Zou GR, Zhang N (2025). Adjuvant PD-1 Blockade With Camrelizumab for Nasopharyngeal Carcinoma. JAMA.

[12] Liu SL, Li XY, Yang JH, Wen DX, Guo SS, Liu LT (2024). Neoadjuvant and adjuvant toripalimab for locoregionally advanced nasopharyngeal carcinoma: a randomised, single-centre, double-blind, placebo-controlled, phase 2 trial. Lancet Oncol.

[13] Cai M, Wang Y, Ma H, Yang L, Xu Z (2024). Advances and challenges in immunotherapy for locally advanced nasopharyngeal carcinoma. Cancer Treat Rev.

[14] Liu X, Yi Y (2020). Recent updates on Sintilimab in solid tumor immunotherapy. Biomark Res.

[15] Zhang L, Mai W, Jiang W, Geng Q (2020). Sintilimab: A Promising Anti-Tumor PD-1 Antibody. Front Oncol.

[16] Ma M, Qi H, Hu C, Xu Z, Wu F, Wang N (2021). The binding epitope of sintilimab on PD-1 revealed by AbMap. Acta Biochim Biophys Sin.

[17] Rajasekaran N, Wang X, Ravindranathan S, Chin DJ, Tseng SY, Klakamp SL (2024). Toripalimab, a therapeutic monoclonal anti-PD-1 antibody with high binding affinity to PD-1 and enhanced potency to activate human T cells. Cancer Immunol Immunother.

[18] Lickliter JD, Gan HK, Voskoboynik M, Arulananda S, Gao B, Nagrial A (2020). A First-in-Human Dose Finding Study of Camrelizumab in Patients with Advanced or Metastatic Cancer in Australia. Drug Des Devel Ther.

[19] Liu K, Tan S, Jin W, Guan J, Wang Q, Sun H (2020). N‐glycosylation of PD‐1 promotes binding of camrelizumab. EMBO Rep.

[20] Lin Y, Lin Y, Zhong X, Chen Q, Tang S, Chen J (2023). A case report and literature review on reactive cutaneous capillary endothelial proliferation induced by camrelizumab in a nasopharyngeal carcinoma patient. Front Oncol.

[21] Lu D, Yao J, Yuan G, Gao Y, Zhang J, Guo X (2022). Immune Checkpoint Inhibitor-related New-onset Thyroid Dysfunction: A Retrospective Analysis Using the US FDA Adverse Event Reporting System. Oncologist.

[22] Liu S, Zhao Q, Zheng Z, Liu Z, Meng L, Dong L (2021). Status of Treatment and Prophylaxis for Radiation-Induced Oral Mucositis in Patients With Head and Neck Cancer. Front Oncol.

[23] King SN, Dunlap NE, Tennant PA, Pitts T (2016). Pathophysiology of Radiation-Induced Dysphagia in Head and Neck Cancer. Dysphagia.

[24] Srivastava A, Nogueras-Gonzalez GM, Geng Y, Singh J, Myers JN, Li Y (2024). Oral Toxicities Associated with Immune Checkpoint Inhibitors: Meta-Analyses of Clinical Trials. J Immunother Precis Oncol.

[25] Edner NM, Carlesso G, Rush JS, Walker LSK (2020). Targeting co-stimulatory molecules in autoimmune disease. Nat Rev Drug Discov.

[26] Xu C, Chen YP, Du XJ, Liu JQ, Huang CL, Chen L (2018). Comparative safety of immune checkpoint inhibitors in cancer: systematic review and network meta-analysis. BMJ.

[27] Francisco LM, Sage PT, Sharpe AH (2010). The PD‐1 pathway in tolerance and autoimmunity. Immunol Rev.

[28] Wang JH (2020). Why the Outcome of Anti‐Tumor Immune Responses is Heterogeneous: A Novel Idea in the Context of Immunological Heterogeneity in Cancers. BioEssays.

[29] National Cancer Institute.

[30] Brahmer JR, Lacchetti C, Schneider BJ, Atkins MB, Brassil KJ, Caterino JM (2018). Management of Immune-Related Adverse Events in Patients Treated With Immune Checkpoint Inhibitor Therapy: American Society of Clinical Oncology Clinical Practice Guideline. J Clin Oncol.

