# Supplementary material for: Addition of immune checkpoint inhibitor to standard of care chemoradiotherapy in patients with locally advanced nasopharyngeal carcinoma: a systematic review and meta-analysis
Source: Explor Target Antitumor Ther. 2026 Sep 28;7:1002403. doi: 10.37349/etat.2026.1002403 (PMC13623625; doi:10.37349/etat.2026.1002403)
Supplement: Supplementary file 1 [file 1002403_sup_1.pdf]

Supplementary Materials:

Table S1. Database search strategies

Database	Boolean search strategy	Limits/date range
MEDLINE	(("Nasopharyngeal Neoplasms"[Mesh] OR nasopharyngeal carcinoma[tiab] OR nasopharyngeal cancer[tiab] OR nasopharynx carcinoma[tiab] OR nasopharynx cancer[tiab] OR NPC[tiab]) AND ("locally advanced"[tiab] OR locoregionally advanced[tiab] OR loco-regionally advanced[tiab] OR nonmetastatic[tiab] OR non-metastatic[tiab] OR stage III[tiab] OR stage IV[tiab] OR stage IVA[tiab] OR stage IVB[tiab]) AND ("Immune Checkpoint Inhibitors"[Mesh] OR "Immunotherapy"[Mesh] OR immunotherapy[tiab] OR immune checkpoint inhibitor*[tiab] OR checkpoint inhibitor*[tiab] OR ICI[tiab] OR ICIs[tiab] OR PD-1[tiab] OR PD1[tiab] OR programmed death 1[tiab] OR PD-L1[tiab] OR PDL1[tiab] OR programmed death ligand 1[tiab] OR anti-PD-1[tiab] OR anti-PD-L1[tiab] OR sintilimab[tiab] OR toripalimab[tiab] OR camrelizumab[tiab]))	Humans; English; database inception to September 1, 2025
EMBASE	('nasopharynx cancer'/exp OR 'nasopharyngeal carcinoma':ti,ab,kw OR 'nasopharyngeal cancer':ti,ab,kw OR 'nasopharynx carcinoma':ti,ab,kw OR 'nasopharynx cancer':ti,ab,kw OR NPC:ti,ab,kw) AND ('locally advanced':ti,ab,kw OR 'locoregionally advanced':ti,ab,kw OR 'loco-regionally advanced':ti,ab,kw OR nonmetastatic:ti,ab,kw OR 'non-metastatic':ti,ab,kw OR 'stage III':ti,ab,kw OR 'stage IV':ti,ab,kw OR 'stage IVA':ti,ab,kw OR 'stage IVB':ti,ab,kw) AND ('immune checkpoint inhibitor'/exp OR 'cancer immunotherapy'/exp OR immunotherapy:ti,ab,kw OR 'immune checkpoint inhibitor*':ti,ab,kw OR 'checkpoint inhibitor*':ti,ab,kw OR ICI:ti,ab,kw OR ICIs:ti,ab,kw OR 'PD-1':ti,ab,kw OR PD1:ti,ab,kw OR 'programmed death 1':ti,ab,kw OR 'PD-L1':ti,ab,kw OR PDL1:ti,ab,kw OR 'programmed death ligand 1':ti,ab,kw OR 'anti-PD-1':ti,ab,kw OR 'anti-PD-L1':ti,ab,kw OR sintilimab:ti,ab,kw OR toripalimab:ti,ab,kw OR camrelizumab:ti,ab,kw) AND [humans]/lim AND [english]/lim	Humans; English; database inception to September 1, 2025

Supplementary Table S1. Database search strategies. Boolean search strings used for MEDLINE and EMBASE to identify randomized controlled trials evaluating immune checkpoint inhibitors in combination with standard chemoradiotherapy-based treatment for locally advanced nasopharyngeal carcinoma. Searches were limited to human studies published in English from database inception through September 1, 2025.
